# Bradykinin‐bradykinin receptor (B1R) signalling is involved in the blood–brain barrier disruption in moyamoya disease

**DOI:** 10.1111/jcmm.17989

**Published:** 2023-10-11

**Authors:** Haidong Wang, Wenxue Sun, Fengfeng Li, Pei Jiang, Lei Wang, Nannan Zhou, Lei Feng

**Affiliations:** ^1^ Department of Pharmacy The Affiliated Lianyungang Hospital of Xuzhou Medical University/The First People's Hospital of Lianyungang/First Affiliated Hospital of Kangda College of Nanjing Medical University Lianyungang China; ^2^ Translational Pharmaceutical Laboratory, Jining First People's Hospital Shandong First Medical University Jining China; ^3^ Institute of Translational Pharmacy Jining Medical Research Academy Jining China; ^4^ Department of neurosurgery, Tengzhou Central People's Hospital Jining Medical University Tengzhou China; ^5^ Department of Neurosurgery, Jining First People's Hospital Shandong First Medical University Jining China

**Keywords:** blood–brain barrier, bradykinin, bradykinin receptor, des‐Arg9‐BK, Moyamoya disease

## Abstract

Moyamoya disease (MMD) is a rare disorder of the cerebrovascular system. It is a steno‐occlusive disease that involves angiogenesis and blood–brain barrier (BBB) disruption. Bradykinin (BK), its metabolite des‐Arg9‐BK, and receptor (B1R) affect angiogenesis and BBB integrity. In this study, we aimed to investigate the changes in BK, B1R and des‐Arg9‐BK levels in the serum and brain tissues of patients with MMD and explore the underlying mechanism of these markers in MMD. We obtained the serum samples and superficial temporal artery (STA) tissue of patients with MMD from the Department of Neurosurgery of the Jining First People's Hospital. First, we measured BK, des‐Arg9‐BK and B1R levels in the serum of patients by means of ELISA. Next, we performed immunofluorescence to determine B1R expression in STA tissues. Finally, we determined the underlying mechanism through Western blot, angiogenesis assay, immunofluorescence, transendothelial electrical resistance and transcytosis assays. Our results demonstrated a significant increase in the BK, des‐Arg9‐BK and B1R levels in the serum of patients with MMD compared to healthy controls. Furthermore, an increase in the B1R expression level was observed in the STA tissues of patients with MMD. BK and des‐Arg9‐BK could promote the migratory and proliferative abilities of bEnd.3 cells and inhibited the formation of bEnd.3 cell tubes. In vitro BBB model showed that BK and des‐Arg9‐BK could reduce claudin‐5, ZO‐1 and occluding expression and BBB disruption. To the best of our knowledge, our results show an increase in BK and B1R levels in the serum and STA tissues of patients with MMD. BK and Des‐Arg9‐BK could inhibit angiogenesis, promote migratory and proliferative capacities of cells, and disrupt BBB integrity. Therefore, regulating BK, des‐Arg9‐BK and B1R levels in the serum and the brain could be potential strategies for treating patients with MMD.

## 1 BACKGROUND

Moyamoya disease (MMD) is a rare disorder of the cerebrovascular system. Progressive narrowing or occlusion of the intracranial internal carotid arteries and their branches are characteristics of MMD. This leads to the formation of moyamoya vessels, which are fragile collateral blood vessels at the base of the brain.^1^, ^2^ The moyamoya vessel means ‘puff of smoke’ in Japanese. These vessels lead to chronic ischemia, haemorrhagic stroke, transient ischemic attack and cognitive impairment. If left untreated, these moyamoya vessels could cause permanent neurological deficits and disabilities. MMD is highly prevalent in Asian populations, specifically in East Asian populations (China, Japan, Korea, etc.). The prevalence of MMD is 0.35–0.54/100,000 individuals per year.^3^ Genetic predisposition, environmental factors and immune factors are associated with MMD pathogenesis; however, the aetiology and pathogenesis of MMD are unclear.

Angiogenesis is the formation of new blood vessels from pre‐existing ones, which is crucially involved in the development and maintenance of the vascular network in the brain.^4^, ^5^ Angiogenesis orchestrates the proliferation, differentiation and migration of endothelial cells (ECs) and pericytes. The migration and invasion of cells are essential steps in angiogenesis as ECs migrate toward the site of angiogenesis and invade the surrounding tissue to form new blood vessels. In addition to physiological angiogenesis, abnormal or pathological angiogenesis has been implicated in various neurological disorders, including MMD. Recent studies have shown that the dysregulation of angiogenic factors, such as vascular endothelial growth factor (VEGF), angiopoietin‐1, transforming growth factor‐beta 1 and platelet‐derived growth factor could cause abnormal angiogenesis in patients with MMD.^6^, ^7^ These angiogenic factors stimulate the proliferative and migratory abilities of ECs and pericytes and promote the production of extracellular matrix (ECM) proteins and new blood vessel formation. However, the underlying mechanisms of the dysregulation of these angiogenic factors and the aberrant angiogenesis in patients with MMD are poorly understood. Interestingly, studies have shown the involvement of bradykinin (BK) metabolism in angiogenesis and cell migration^8^, ^9^; thus, BK could be involved in MMD pathogenesis.

BK is a nine‐amino acid peptide found in several cells and tissues involved in various physiological and pathological processes. BK binds to B1 and B2 (B1R and B2R), G‐protein‐coupled receptors, widely expressed by different cell types to exert its effects. B1R is primarily expressed under inflammatory conditions, whereas B2R is constitutively expressed.^10^, ^11^ BK activates ECs to increase VEGF expression, thereby promoting the migration and proliferation of ECs.^12^ Additionally, BK activates smooth muscle cells and fibroblasts to secrete VEGF and matrix metalloproteinases (MMPs), which facilitate the remodelling and maturation of vessels. Des‐Arg9‐BK is a BK metabolite that impacts biological processes, such as angiogenesis and cell migration.^13^ BK is cleaved by the angiotensin‐converting enzyme or aminopeptidases to generate des‐Arg9‐BK. Furthermore, des‐Arg9‐BK has a higher affinity to the B1R compared to B2R and is primarily involved in inflammatory conditions.^14^ Recent studies have shown that BK and des‐Arg9‐BK are involved in angiogenesis and cell migration. BK enhances the proliferative and migratory ability of ECs and stimulates new blood vessel formation in vivo. Des‐Arg9‐BK enhances angiogenesis under inflammatory conditions and the migration as well as invasion of cancer cells.^15^, ^16^

Moreover, MMD affects the blood–brain barrier (BBB), a complex network of specialized cells essential for maintaining a stable environment for the brain. BBB protects the brain from harmful substances in the blood.^17^ BBB disruption occurs in patients with MMD, which increases the permeability and susceptibility to neurotoxic insults. The disruption of BBB facilitates the proinflammatory cytokines, immune cells and factors to enter the brain, which damages the brain and contributes to MMD pathogenesis. Studies have explored the role and mechanism of the BK/B1R in BBB, which revealed that BK could cause injury to BBB. Interestingly, the knockdown of B1R could exert beneficial effects.^18^, ^19^

Multiple studies have explored the mechanism of MMD pathogenesis; however, no study has investigated the correlation between BK and MMD. Therefore, in this study, we aimed to investigate BK, B1R and des‐Arg9‐BK levels in the serum of patients with MMD. Next, we determined the effect of BK, B1R and des‐Arg9‐BK on angiogenesis and BBB integrity. Our results could shed light on MMD pathogenesis and the potential of BK as a potential target for treating patients with MMD.

## 2 MATERIALS AND METHODS

### 2.1 Study population

We enrolled patients with MMD receiving treatment at the Department of Neurosurgery of the Jining First People's Hospital. Patients with MMD were diagnosed based on strict diagnostic criteria outlined by the Research Committee on Spontaneous Occlusion of the Circle of Willis of the Ministry of Health, Labor, and Welfare in Japan. The diagnostic criteria included severe occlusion or stenosis of the internal carotid and middle as well as anterior cerebral arteries along with an atypical collateral vessel network.^20^, ^21^ The patients were excluded based on the following criteria: patients with secondary MMD due to atherosclerosis, hyperthyroidism, neurofibromatosis, meningitis, leptospiral infection or prior skull‐base radiation therapy. Healthy controls (HCs) recruited from hospitals were age‐ and sex‐matched. All experiments including human participants were performed in accordance with the Helsinki Declaration and were approved by the medical ethics committee of the Jining First People's Hospital. All participants or their guardians provided informed consent.

### 2.2 Sample collection

We collected small fragments of superficial temporal artery (STA) tissue during the surgery. We collected blood from patients before and during the surgery using lithium heparin‐containing tubes and centrifuged the blood samples. Next, we collected and stored the serum at −80°C for future analysis. We fixed vascular tissues in 4% paraformaldehyde (PFA) for 24 h, embedding them in paraffin and sectioning them for further examination.

We obtained and stored the serum of participants from the HC group following the same method. These experiments were performed following established protocols to ensure the accuracy and reliability of the collected samples.

### 2.3 Determining BK, des‐Arg9‐BK and B1R levels in the serum

Serum levels of BK (CSB‐E09155h, Cusabio), B1R (CSB‐EL002651HU, Cusabio) and des‐Arg9‐BK (HB‐PD6317S, Huabang Bio) were quantified by the Quantikine enzyme‐linked immunosorbent assay (ELISA) kits based on the protocol provided by the manufacturer. Briefly, we added serum samples and standards to antihuman monoclonal antibodies‐coated wells, sealed the plates with a membrane and incubated them at 37°C for 30 min. Next, we added a secondary horseradish peroxidase (HRP)‐labelled antibody to all wells and incubated them for an antigen–antibody‐HRP complex formation. Furthermore, we washed the wells, added the substrate solutions A and B, and incubated them for 10 min. The reaction was then stopped, resulting in a yellow colour. All analyses were performed in duplicates, and the results were averaged.

### 2.4 Cell culture and treatment

In this study, we collected immortalized mouse brain endothelial cells (bEnd.3 cells) obtained from ATCC (Manassas, VA, USA), and cultured them in Dulbecco's modified Eagle's medium (DMEM) supplemented with 10% fetal bovine serum (FBS), 100 units/mL penicillin, and 100 μg/mL streptomycin at 37°C and 5% CO_2_ until confluent. DMEM contained 4500 mg/L D‐glucose, 110 mg/L sodium pyruvate, L‐glutamine and 1.5 g/L sodium bicarbonate. Once cells were confluent, they were treated with 100 nM BK or des‐Arg‐BK for 2 h.

### 2.5 Immunofluorescence (IF)

For antigen retrieval, we deparaffinized the tissue sections with xylene and hydrated them with descending gradients of ethanol. We fixed and permeabilized bEND.3 cells with ice‐cold methanol and acetone, respectively.

The slides containing tissue sections were blocked with 5% donkey serum in TBS solution (Servicebio) and incubated with CD31 (Abcam, ab9498), B1R (LSBio, LC‐C196744‐100), Claudin‐5 (Abcam, ab131259), occludin (Abcam, ab216327) and ZO‐1 (Abcam, ab307799) primary antibodies overnight at 4°C. Next, the tissues were incubated with corresponding fluorescent secondary antibodies (Cell Signalling Technology) for 2 h at room temperature, washed with TBS thrice, incubated with DAPI (Servicebio) for 30 min and mounted in glycerol (Servicebio). We observed the IF signals using an Olympus Fluoview laser scanning confocal microscope (Olympus Corporation, Tokyo, Japan). We counted the percentage of positive cells using the ‘ImageJ 1.8.0’ software (National Institutes of Health, MD, USA).

### 2.6 Evaluation of blood–brain barrier in vitro model‐transendothelial electrical resistance (TEER) and transcytosis assays

We seeded the cells on 0.4 μm pore size inserts in a 24‐well transwell plate (Corning) for 1 week to establish a blood–brain barrier model in vitro,^22^ and we evaluated its integrity by the measurement of TEER values daily as previously described.^23^ The total resistance (Ω) was measured using an STX2 probe of the EVOM2 system. We normalized all TEER values to the area of the membrane, and the values were corrected for the resistance without cells. We performed all experiments in duplicates and a minimum of three independent differentiations. We plotted TEER versus time graphs for normalizing TEER values to the peak value of the control, such that the maximum relative TEER value of each biological replicate of the control reached 1.0 at its highest value. A TEER value greater than 200 Ω*cm^2^ indicated the successful establishment of the model, allowing for subsequent drug administration experiments.

The permeability of BBB model was assessed once the cell monolayer had formed a dense layer. In the upper chamber of the transwell insert, we added 0.1 mL of a solution containing 0.5 mg/mL FITC‐dextran (prepared in serum‐free, phenol red‐free DMEM), while in the lower chamber, we added 0.6 mL of a solution containing 0.5 mg/mL non‐FITC‐labelled dextran (also prepared in serum‐free, phenol red‐free DMEM). After incubating in the cell culture incubator for 1 h, we separately extracted samples from the upper and lower chambers of the transwell device for fluorescence intensity measurements. The permeability coefficient (Pd) of the cell layer was calculated using the following formula: Pd (cm/s) = (C/t) × (1/A) × (V/L).

Where: C represents the concentration of FITC‐dextran in the lower chamber, t is the time interval, A is the surface area, V is the volume of the lower chamber solution and L is the concentration of FITC‐dextran in the upper chamber.

### 2.7 Angiogenesis assay

In conditioned media, endothelial cells respond to angiogenic signals by forming capillary‐like structures. The process of tube formation occurs rapidly, with endothelial cells aligning themselves and lumen‐containing tubules beginning to appear within a remarkably short timeframe. The tube formation assay was proposed as a rapid, reproducible and highly sensitive in vitro method for measuring angiogenesis.^24^ First, cells were treated, and bEnd.3 cells were seeded at a density of 1 × 10^5^ cells/well in a 24‐well plate coated with Matrigel and incubated for 8 h under normal conditions. Next, we selected and imaged five representative fields. For quantitation, total tube length and number of tube branches were calculated using ImageJ software. All experiments were performed in triplicate and analysed blindly.

### 2.8 Migration assay

For the migration assay, we seeded bEnd.3 cells on the upper chamber of a transwell membrane in 24‐well plates for 24 h. Next, cells migrated to the lower chamber were fixed, stained and quantified with the aid of a microscope. All experiments were performed in triplicate.

### 2.9 EdU test

We performed the EdU test to determine the effect of the BK and des‐Arg9‐BK on the proliferation of bEnd.3 cells. Briefly, 1 × 10^5^ cells were cultured in DMEM+10% FBS for 24 h. First, we incubated the cells with EdU‐labeling reagent (1:1000, Invitrogen) for 2 h, discarded the medium, and washed the cells with phosphate‐buffered saline (PBS) twice for 5 min. Next, we fixed the cells with 4% PFA and incubated them with 2 mg/mL of glycine for 5 min, respectively. The cells were rinsed with PBS for 5 min and stained with the Click‐iT Edu Alexa Fluor 555 Imaging Kit (Invitrogen) and DAPI (1:500, Invitrogen) for 5 min. Finally, we examined the nuclei using a fluorescence microscope and determined the count of EdU+ cells.

### 2.10 Western blot

First, we extracted and quantified total cellular protein using a BCA protein assay kit (Beyotime, Shanghai, China). Next, we separated proteins on 10% SDS/PAGE and transferred them onto a polyvinylidene difluoride membrane (Millipore, USA). The proteins were blocked with 5% skimmed milk for 1 h, incubated with primary rabbit occludin (Abcam, #ab216327), claudin‐5 (Abcam, #ab131259), ZO‐1 (Abcam, #ab307799) (all antibodies were diluted to 1:1000) and β‐actin (1:2000; Cell Signalling Technology, 4970 s) polyclonal antibodies overnight at 4°C, and washed with PBS. Next, the membranes were incubated with an antimouse HRP‐conjugated secondary antibody (Affinity Biosciences, S0001,) (1:2000) for 1 h. Finally, the membranes were visualized using an ECL kit (Beyotime, China), and β‐actin served as a reference control.

### 2.11 Statistical analysis

We statistically analysed the data using the Statistical Package for Social Sciences (SPSS) version 19.0 software (SPSS Inc., Chicago, IL, USA). Next, an independent *t*‐test was employed to compare the baseline levels of the two groups for normally distributed variables. Variables were compared using a *t*‐test or Mann–Whitney U test as appropriate to determine the presence of MMD, and multiple logistic regression analysis (MLRA) was conducted. We performed receiver operating characteristic (ROC) curve analysis to split continuous variables using a calculated cut‐off value. All histograms were plotted using GraphPad Prism 9.0. *p* < 0.05 was considered statistically significant.

## 3 RESULTS

### 3.1 Demographics of subjects

We enrolled 51 patients with MMD (26 males and 25 females) and 65 matched HCs (39 males and 26 females). Table 1 shows detailed demographic characteristics, including gender, age (in years), body mass index (BMI) and total number of participants. The results showed no significant difference in age, sex and BMI of patients with MMD and HC (*p* > 0.05). The situation for the type of onset and the pathogenic site were summarized for patients in the MMD group.

**TABLE 1:** Demographic and clinical characteristics of the study subjects.

Variables	Controls (*n* = 65)	MMD (*N* = 51)	*p* Value
Age (years)	44.17 ± 9.19	46.31 ± 12.62	0.29
Gender (M/F, *n*)	39/26	26/25	0.331
BMI (kg/m^2^)	23.74 ± 4.10	24.28 ± 3.21	0.44
Type of onset (*n*, %)			
Infarction		47 (92.16)	NA
Haemorrhage		4 (7.84)	
Pathogenic site (*n*, %)			
Bilateral		42 (82.35)	NA
Unilateral		9 (17.65)	

Abbreviation: MMD, moyamoya disease.

### 3.2 BK, des‐Arg9‐BK and B1R levels in the serum of patients with MMD and MLRA to predict MMD risk

Compared to HC, we observed a significant increase in BK (11.54 ± 2.88 μg/mL vs. 9.75 ± 3.10 μg/mL, *p* = 0.002, 1a‐1c), des‐Arg9‐BK (0.57 ± 0.12 μg/L vs. 0.45 ± 0.13 μg/L, *p* < 0.001) and B1R (102.44 ± 26.09 pg/mL vs. 88.09 ± 22.09 pg/mL, *p* = 0.0023) levels were observed in the serum of patients with MMD.

**FIGURE 1:**
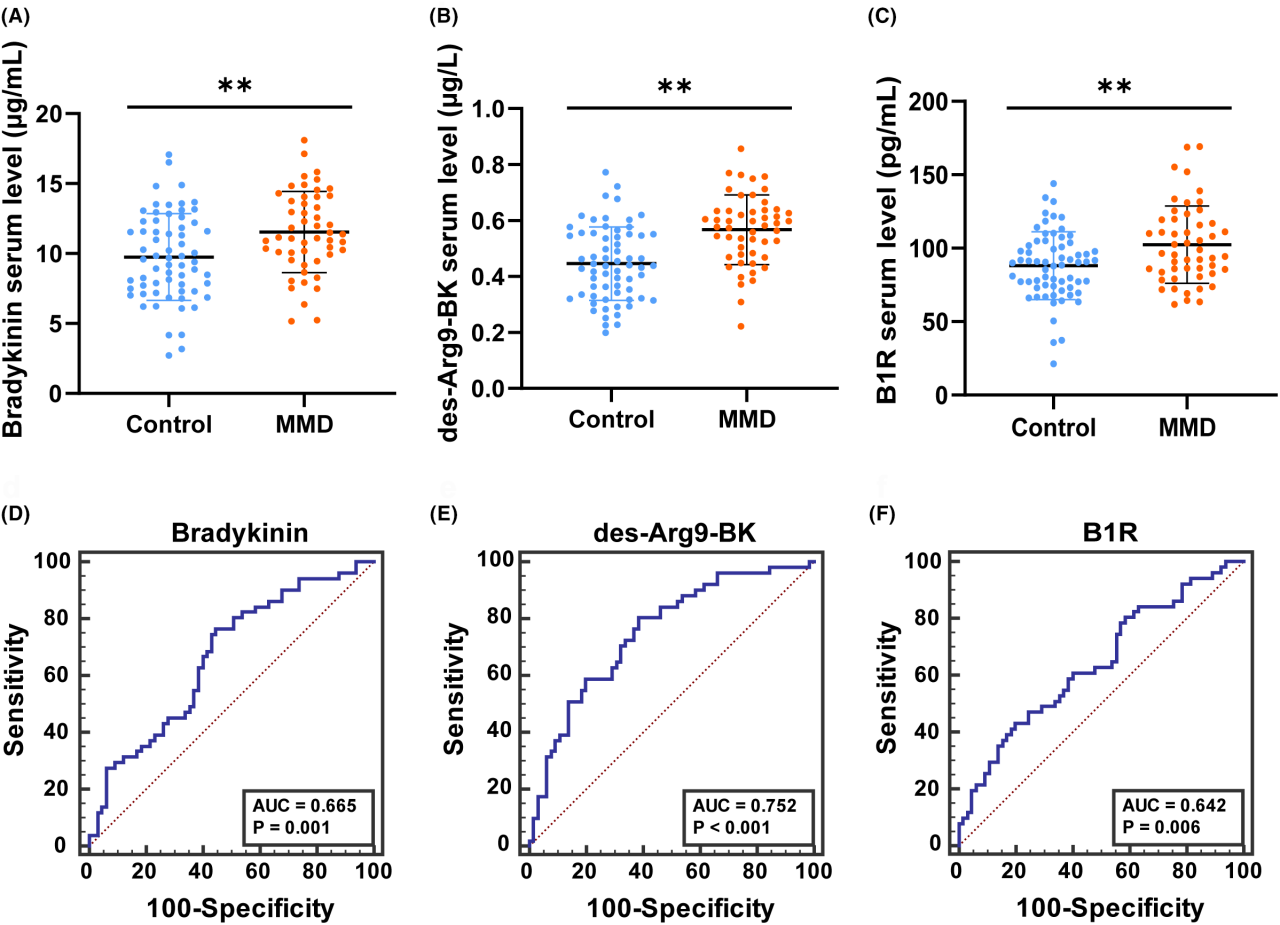
Levels of Bradykinin (BK) (A), Des‐Arg9‐BK (B) and B1R (C) in the serum of patients with MMD and healthy subjects. Data are expressed as the mean ± SD. **p < 0.01. ROC curve analyses of the Bradykinin (BK) (D), Des‐Arg9‐BK (E) and B1R (F) ROC, receiver operating characteristic; AUC, area under the curve.

MLRA was conducted to determine if BK, des‐Arg9‐BK and B1R could predict MMD risk. We dichotomized the continuous variables based on a cut‐off value calculated using the ROC curve. The area under the ROC curve value of BK was 0.665 (*p* = 0.001), des‐Arg9‐BK was 0.752 (*p* < 0.001) and B1R was 0.642 (*p* = 0.006, Figure 1D,F).

MLRA revealed that decreased BK (odds ratio (OR): 3.745, 95% confidence interval (CI): 1.551–9.041; *p* = 0.003), des‐Arg9‐BK (OR: 5.382, 95% CI: 2.172–13.336; *p* = 0.002), together with B1R (OR: 2.798, 95% CI: 1.102–7.102; *p* = 0.030, Table 2). This suggests that BK, B1R and des‐Arg9‐BK could predict MMD risk.

**TABLE 2:** Logistic regression analysis for the presence of MMD.

Variables	OR (95% CI)	*p* Value
Bradykinin	3.745 (1.551–9.041)	0.003
des‐Arg9‐BK	5.382 (2.172–13.336)	0.002
B1R	2.798 (1.102–7.100)	0.030

Abbreviations: 95% CI, 95% confidence intervals; OR, odds ratio.

### 3.3 Increase in B1R expression in the STA tissues of patients with MMD

**FIGURE 2:**
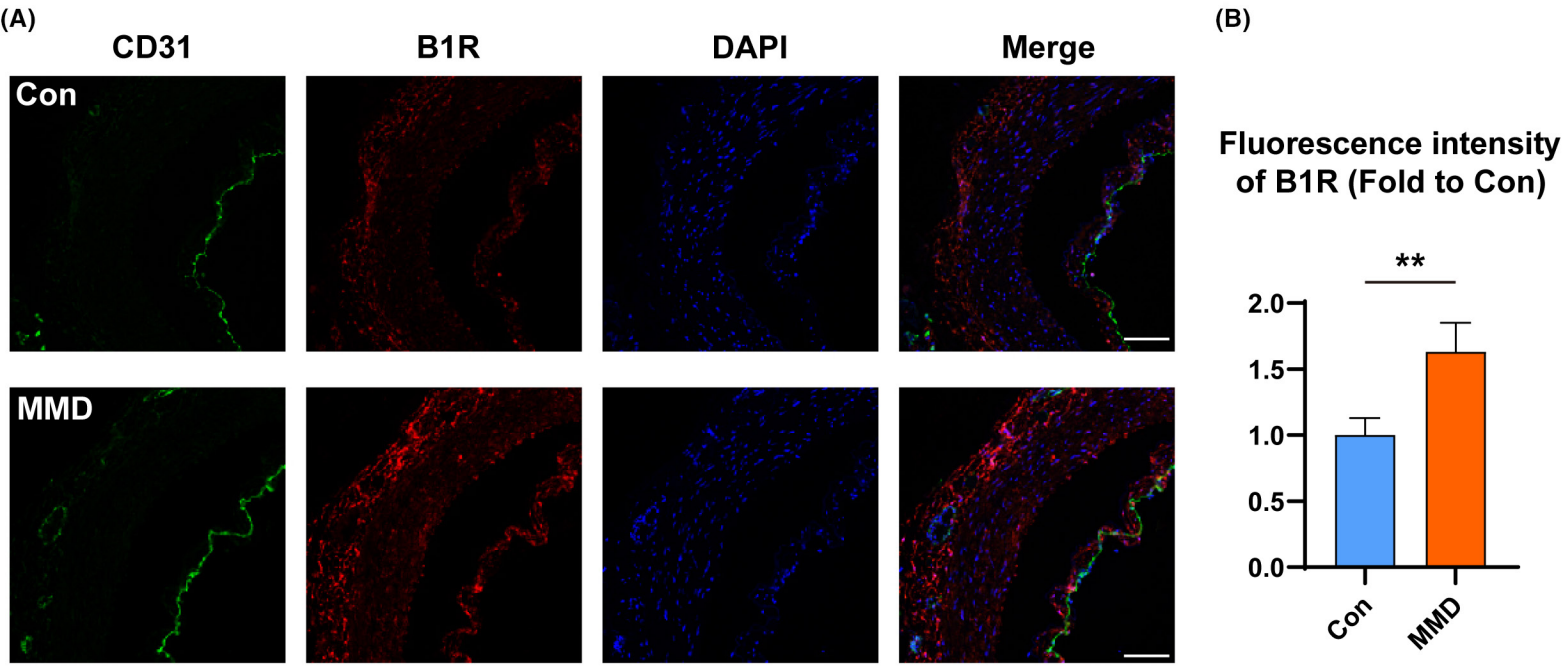
Immunofluorescence staining assay for B1R and CD31 expression on the superficial temporal artery (STA) tissue section from patients with MMD and healthy subjects. A: Green CD31, Red B1R, Blue DAPI, Scale: 50 μm. (*n* = 6) B: Fluorescence intensity of B1R in MMD patients (fold to the control) (*n* = 6) **p < 0.01.

We measured B1R expression by IF (Figure 2). The results revealed an increase in B1R expression in STA tissues of patients with MMD compared to HCs, consistent with quantitative analysis in the serum of the participants. B1R intensity in the STA tissues of patients with MMD was approximately 1.6 times higher compared to HCs.

### 3.4 BK and des‐Arg9‐BK Limit bEnd.3 Cell angiogenesis

**FIGURE 3:**
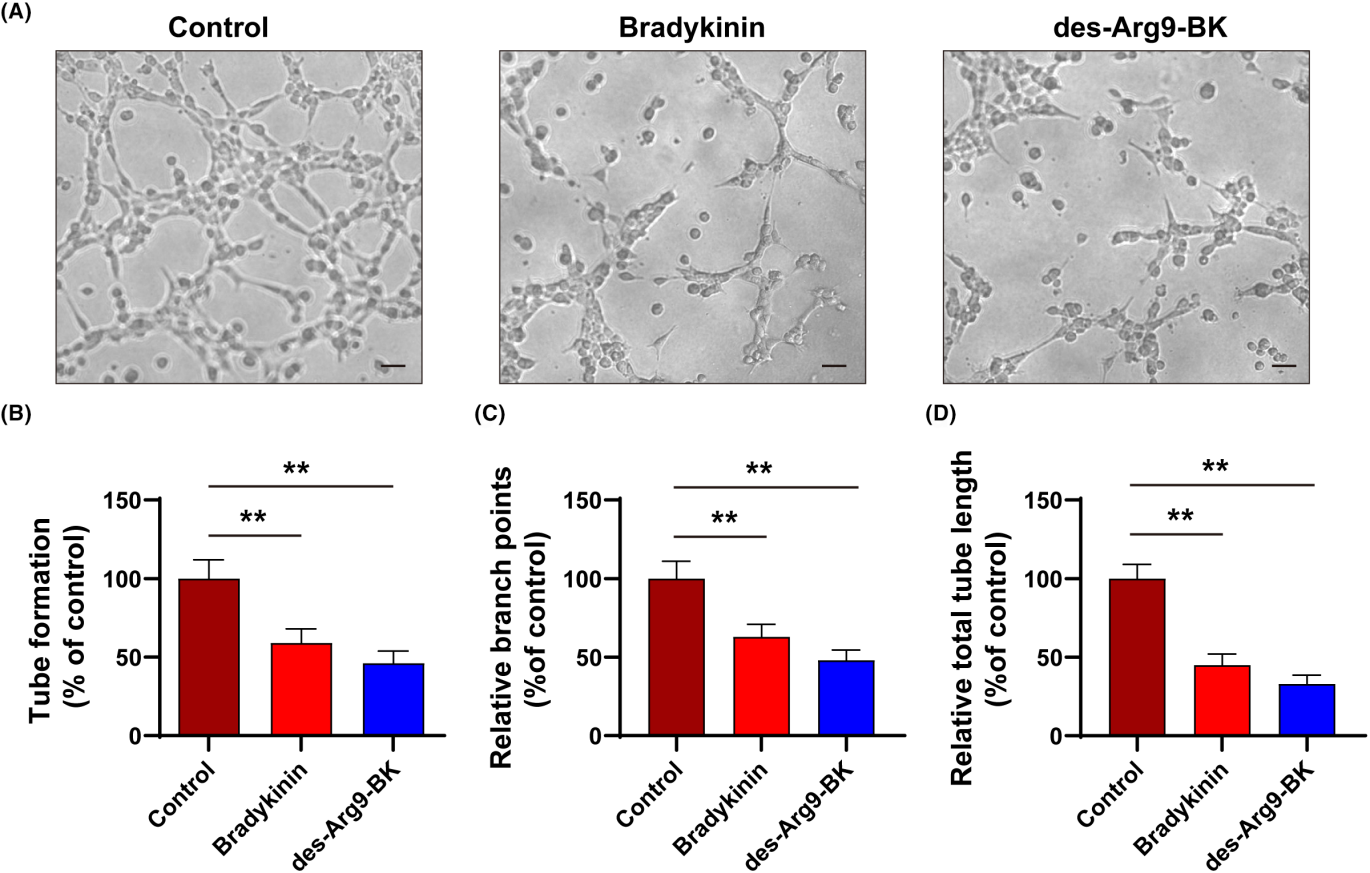
Bradykinin (BK) and Des‐Arg9‐BK Limit Tube Formation bEnd.3 cells. Representative images (A–C), Scale: 100 μm. Tube formation rate (D), relative branch points (E) and relative total tube length cultured with BK and des‐Arg9‐BK compared with controls **p < 0.01.

BEnd.3 cells cultured on 3D ECM form capillary‐like structures, or tubes, thus indicating the ability of cells to form the inner lining of blood vessels, recapitulates several angiogenesis features in vivo.^25^ Hence, we cultured bEnd.3 cells with BK and des‐Arg9‐BK individually to observe the effects of BK and des‐Arg9‐BK on cell angiogenesis. Figure 3A shows cell angiogenesis under different culture conditions.

Next, we quantified the angiogenesis parameters, including the rate of formation, branching and length of tubes (Figure 3B–D). The angiogenesis parameters of cells cultured with BK and des‐Arg9‐BK were significantly lower compared to the control group, indicating that BK and des‐Arg9‐BK could limit the formation of bEnd.3 cell tubes. These results suggest that bEnd.3 cells treated with des‐Arg9‐BK and BK could significantly inhibit angiogenesis.

### 3.5 BK and des‐Arg9‐BK enhance the migratory and proliferative capacities of bEnd.3 cells

**FIGURE 4:**
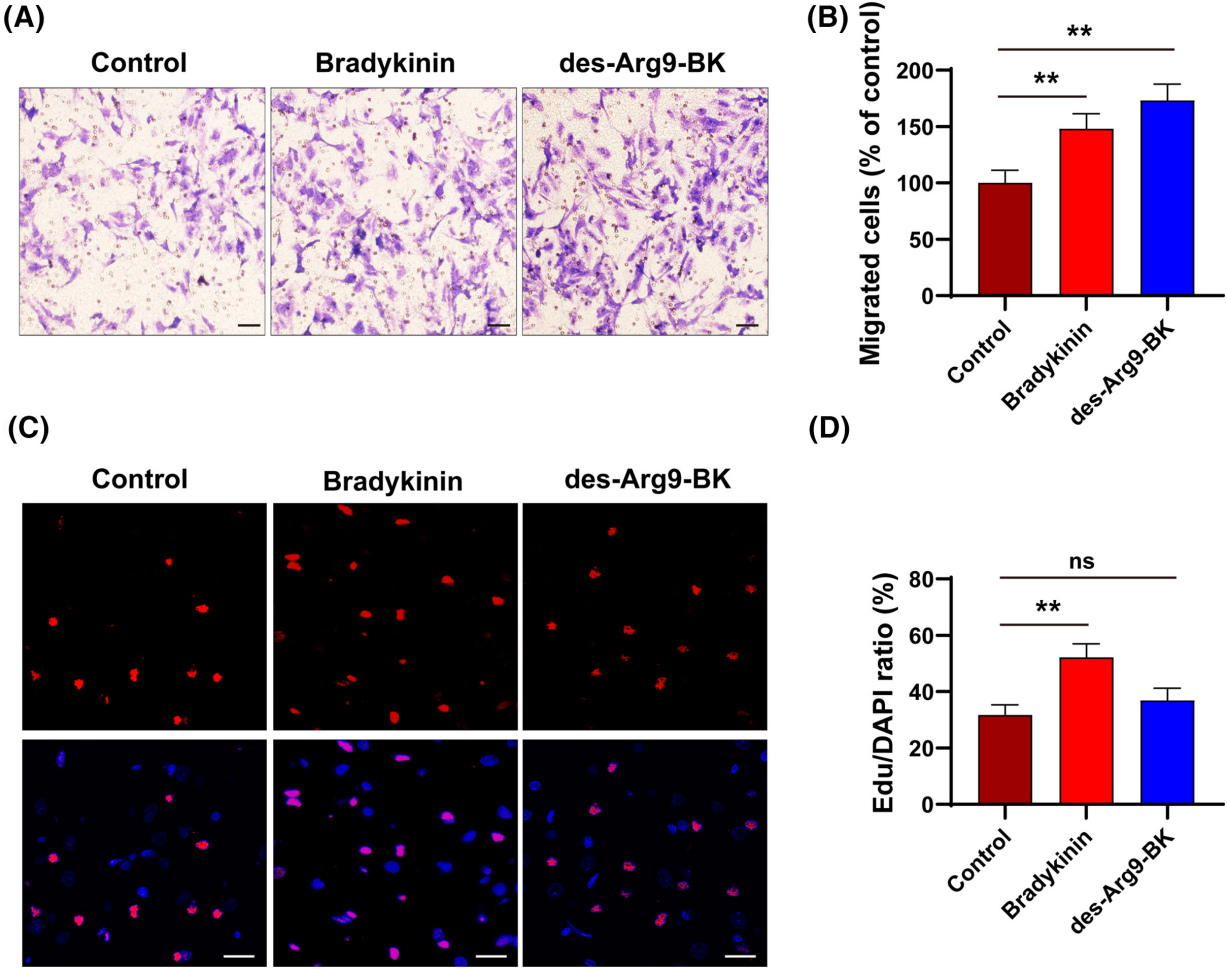
Bradykinin (BK) and Des‐Arg9‐BK promote bEnd.3 cells migration and proliferation. (A,B) Transwell assay was performed to evaluate cell migration, Scale: 100 μm; the number of migrated cells was recorded compared with controls. (*n* = 3) (C,D) Cell proliferation was measured via EdU staining, Scale: 20 μm; Quantification of Edu/DAPI compared with controls based on the immunofluorescent images. (*n* = 3). **p < 0.01 ns, not significant.

We determined the effects of BK and des‐Arg9‐BK on the migratory and proliferative capacities of bEnd.3 cells (Figure 4). First, we analysed cell migration using a transwell assay. The results revealed a 140% and 168% increase in the migration of BK and des‐Arg9‐BK‐treated bEnd.3 cells, respectively, compared to cells treated with vehicle control (Figure 4B). Next, we performed the EdU cell proliferation assay to determine the effect of these factors on cell proliferation (Figure 4C,D). The result showed a significant increase in the proliferative ability of BK‐treated bEnd.3 cells and a nonsignificant decrease in the proliferation ability of des‐Arg9‐BK‐treated bEnd.3. This indicates that BK could enhance the migratory and proliferative capacities of bEnd.3 Cells.

### 3.6 BK and des‐Arg9‐BK could disrupt the BBB

**FIGURE 5:**
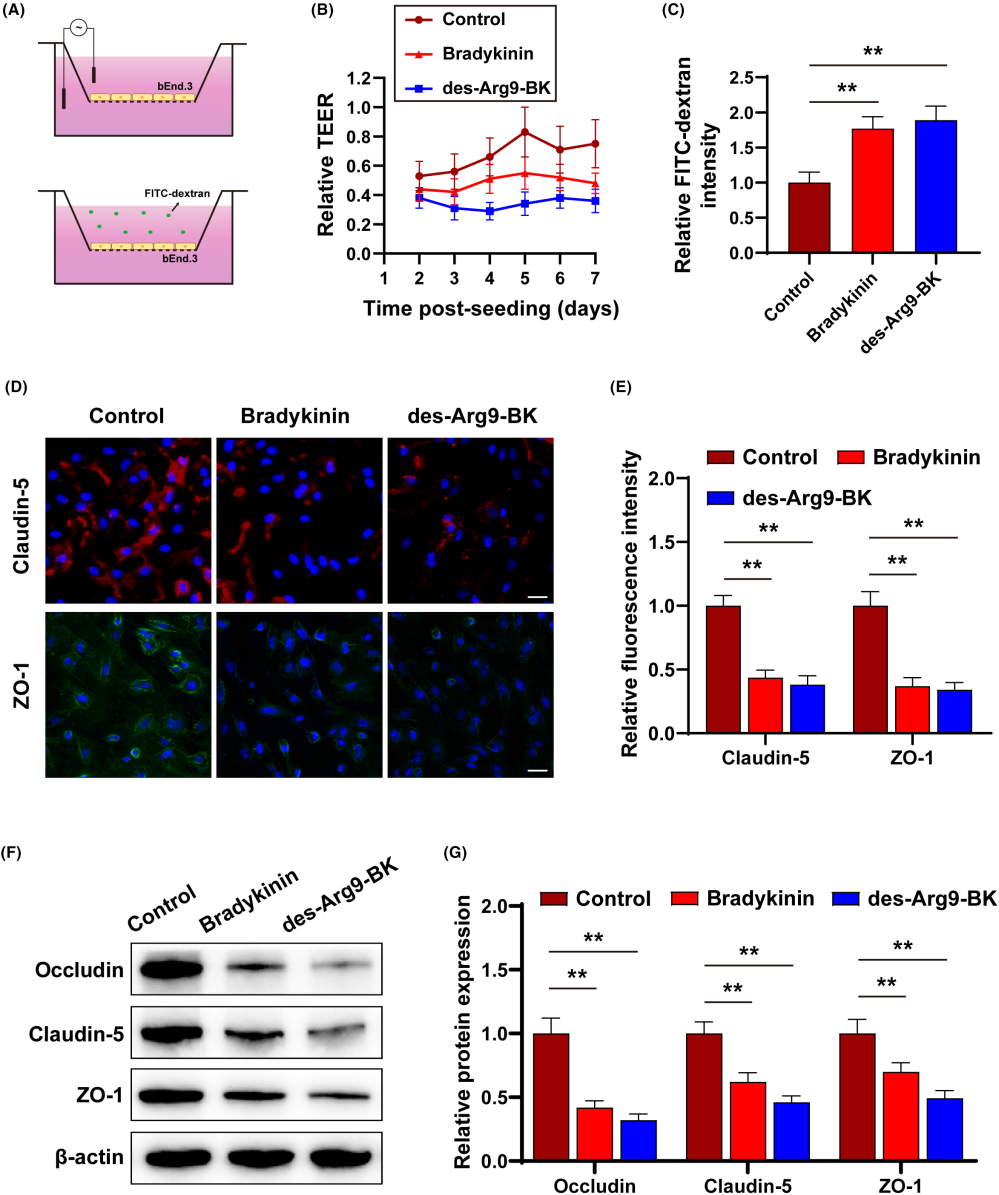
Bradykinin (BK) and Des‐Arg9‐BK distrupt BBB barrier permeability in vitro.s. (A) Pictorial representation of TEER and FITC‐dextran translocation assays. (B) Relative TEER values in different culture through the time postseeding. (C) FITC‐dextran permeability across the bEnd.3 cells. (D) Immunofluorescence staining for Claudin‐5 and ZO‐1 in cultured Bradykinin (BK), Des‐Arg9‐BK and control. (E) Representative Fluorescence intensity of Claudin‐5 and ZO‐1 in cultured Bradykinin (BK), Des‐Arg9‐BK and control. (F) Representative Western blot of Occludin, Claudin‐5 and ZO‐1 in cultured Bradykinin (BK), Des‐Arg9‐BK and control. (G) Quantification of Occludin, Claudin‐5 and ZO‐1 in cultured Bradykinin (BK), Des‐Arg9‐BK and control, respectively.

We established an in vitro BB model by culturing bEnd3 to observe the effects of BK and des‐Arg9‐BK on the integrity and permeability of BBB (Figure 5A) and determined the changes in the integrity of the BBB by measuring TEER and FITC‐dextran. The results revealed a significant decrease in the relative TEER value of the cells exposed to BK or des‐Arg9‐BK compared to control cells (Figure 5B). This indicates that BK and des‐Arg9‐BK could disrupt the BB. Similarly, the FITC‐dextran results showed a significant increase in the relative intensity of FITC‐dextran in BK and des‐Arg9‐BK‐treated cells compared to control cells (Figure 5C), thus indicating BK and des‐Arg9‐BK could increase the permeability of BBB. Next, we determined the expression and distribution of tight junction (TJ) proteins, including claudin‐5, ZO‐1 and occludin using IF (Figure 5D,E) and Western blot (Figure 5F,G). The results demonstrated a significant decrease in TJ protein expression level in those treated with BK and des‐Arg9‐BK‐treated bEnd.3, thus demonstrating BBB disruption.

## 4 DISCUSSION

Our results demonstrated an increase in BK, des‐Arg9‐BK and B1R levels in the serum of patients with MMD. ROC curve showed that BK, des‐Arg9‐BK and B1R could predict MMD risk. Moreover, an increase in B1R expression was observed in the STA tissues of patients with MMD. In vitro studies showed that BK and des‐Arg9‐BK could inhibit angiogenesis and enhance the migratory and proliferative capacities of bEnd.3 cells. Lastly, we demonstrated the effects of BK and des‐Arg9‐BK on disrupting the BBB in vitro.

BK causes the vasodilatation of arteries and arterioles via the endothelium in the brain and increases the permeability of the cerebrovascular system, thus causing vasogenic brain edema.^26^, ^27^ Des‐Arg9‐BK is an active BK metabolite that affects single pial venular capillaries. B1R and B2R are BK receptors activated by BK and des‐Arg9‐BK, respectively. Several studies have determined the correlation between BK, BK receptors and cerebrovascular occlusive diseases, such as cerebral ischemia, stroke and cerebral infarction.^26^, ^28^, ^29^ To the best of our knowledge, no studies have demonstrated a correlation between BK, BK receptors or des‐Arg9‐BK and MMD pathogenesis. Our results revealed a significant increase in BK, B1K and des‐Arg9‐BK levels in the serum of patients with MMD compared to HCs, thus suggesting BK, B1K and des‐Arg9‐BK could be involved in MMD pathogenesis. Next, IF results showed a significant increase in B1R expression levels in the STA tissues of patients with MMD, thus indicating the involvement of B1R in MMD pathogenesis. Previous studies have shown that BK and B1R could promote the release of proinflammatory cytokines in the microvascular endothelial cells of the brain^18^, ^30^ and B1R activation induced inflammation in human brain microvascular endothelial cells.^29^ We have previously demonstrated a significant increase in interleukin‐1β, tumour necrosis factor and interleukin‐12 levels in the serum of patients with MMD compared to HCs.^31^ Therefore, the present study is an extension of our previous study to explore additional mechanisms involved in MMD pathogenesis.

Studies have used immunohistochemistry to detect CD31 expression for demonstrating the presence of endothelial cell tissue and evaluating angiogenesis.^32^ Our IF results showed an increase in B1R expression levels in the STA tissues of patients with MMD. These results indicate that B1R and their upstream BK and BK metabolites could affect MMD pathogenesis. Therefore, we established an in vitro model to verify our hypothesis.

BK and des‐Arg9‐BK demonstrated significant inhibitory effects on the formation, branching and length of the vessel in vitro, thus indicating a significant inhibitory effect of BK and des‐Arg9‐BK on angiogenesis. These results strongly support our initial hypothesis. Previous studies have reported that BK and des‐Arg9‐BK regulate angiogenesis. However, there are discrepancies between previous studies and our results. Wang et al. showed that BK promotes angiogenesis.^33^ Additionally, another study demonstrated that BK increases vascular permeability during the early phase via B2R in the endothelial cells to promote angiogenesis.^34^ Moreover, BK promotes angiogenesis and the growth of tumours in vivo.^35^ Guo et al. demonstrated the molecular mechanisms mediating the proangiogenic activity of BK. The use of different cell types in these studies could be the underlying cause of discrepancies in results.^36^ Hence, further studies are required to determine the effect of BK on angiogenesis.

Moreover, our results showed that BK could enhance the migratory and proliferative capacities of cells, consistent with previous studies using different cells. Studies have reported that BK stimulates or promotes the migration and proliferation of gastric cancer, human cardiac progenitor and cervical cancer cells. Further, BK regulated the migratory and proliferative capacities of cells via the ERK and STAT3 signalling pathways.^37^, ^38^ These results shed light on the significance of BK in MMD pathogenesis.

BBB plays a crucial role in the maintenance of homeostasis in the brain. Numerous studies have investigated the correlation between BBB disruption and MMD pathogenesis. A study used sodium fluorescein videoangiography to demonstrate BBB impairment in patients with MMD.^17^ Moreover, Lu et al. showed a significant decrease in cortical perfusion in patients with MMD and disrupted BBB compared to patients with intact BBB. Furthermore, significant disruption of the BBB was observed in patients with MMD compared to patients with arteriosclerotic cerebrovascular disease.^39^ Studies have investigated the application of several biomarkers, including VEGF, MMPs, claudin 5, occludin and junction‐associated molecule‐1, for assessing BBB function in patients with MMD. In this study, we investigated the effects of BK and des‐Arg9‐BK on BBB integrity and the regulation of TJ proteins to determine the role of BK and des‐Arg9‐BK in the pathogenesis of MMD. Our results revealed that BK and des‐Arg9‐BK could disrupt BBB integrity and decreases TJ protein expression levels. Together, these findings indicate that BK and des‐Arg9‐BK could be involved in MMD development by impairing BBB integrity.

However, the present study has a few limitations. First, our sample size was relatively small, and the cross‐sectional study failed to determine a causal correlation. Hence, additional large sample‐size longitudinal studies are required to validate our results. Second, efficient MMD animal models are lacking. Hence, we performed in vitro studies to determine the mechanism underlying the effect of BK and des‐Arg9‐BK on MMD. Additional studies using animal models or human specimens are required to determine the mechanism of BK and des‐Arg9‐BK in patients with MMD. Finally, the correlation between MMD pathogenesis, the proliferation and migration of cells, angiogenesis and BBB impairment should be further explored. These efforts would provide additional evidence to support our findings.

## 5 CONCLUSION

In this study, we demonstrated an increase in BK, des‐Arg9‐BK and B1R levels in the serum and the STA tissues of patients with MMD, thus indicating that these factors could contribute to MMD pathogenesis. Our results showed that BK and des‐Arg9‐BK could inhibit angiogenesis, promote the proliferation and migration of cells and disrupt BBB integrity. Thus, regulating the BK, des‐Arg9‐BK and B1R levels in the serum and the brain could be potential strategies for treating patients with MMD.

## AUTHOR CONTRIBUTIONS

**Haidong Wang:** Formal analysis (equal); writing – original draft (equal). **Wenxue Sun:** Formal analysis (equal); software (equal). **Fengfeng Li:** Conceptualization (equal); supervision (equal). **Pei Jiang:** Funding acquisition (lead); resources (equal); supervision (equal). **Lei Wang:** Investigation (equal); software (equal). **Nannan Zhou:** Data curation (equal); writing – review and editing (equal). **Lei Feng:** Conceptualization (lead); project administration (equal).

## FUNDING INFORMATION

The study was supported by the National Natural Science Foundation of China (P. Jiang, 81,602,846; P. Jiang, 82,272,253); Natural Science Foundation of Shandong Province (ZR2021MH145); Taishan Scholar Project of Shandong Province (tsqn201812159); Traditional Chinese Medicine Science and Technology Development Plan of Shandong Province (M‐2022066) and China International Medical Foundation (No. Z‐2018‐35‐2002).

## CONFLICT OF INTEREST STATEMENT

The authors confirm that there are no conflicts of interest.

## Data Availability

All data included in this study are available upon request by contact with the corresponding author.
